# Understanding women’s suffering and psychological well-being: exploring biopsychosocial factors in mothers of children with ADHD – a case study

**DOI:** 10.3389/fpubh.2023.1279499

**Published:** 2023-10-17

**Authors:** Jonah Angeline, Maya Rathnasabapathy

**Affiliations:** School of Social Sciences and Languages, Vellore Institute of Technology, Chennai, Tamil Nadu, India

**Keywords:** ADHD, parenting stress, psychological well-being, mothers, sufferings of women, women violence

## Abstract

**Overview:**

Parents play a dynamic part in child development. Mothers have a great responsibility towards the upbringing child. Previous research has demonstrated that parenting stress levels are high among Parents of children with attention deficit hyperactivity disorder (ADHD).

**Aim:**

Parenting stress affects the psychological well-being of the mother. It is necessary to identify the factors that affect psychological well-being based on Biopsychosocial factors, including biological, psychological, and social factors.

**Methodology:**

An in-depth case study was conducted with the mother of a 7-year-old child diagnosed with attention deficit hyperactivity disorder.

**Results:**

The mother lacks self-efficacy and low perceived social support in. Equipping themselves and building up the knowledge on handling the child and training the child with the developmental disorder increase parenting self-efficacy. Support from family, partner, and society will help them strengthen themselves and may have high psychological support.

## 1. Introduction

Being a parent is the most significant responsibility a person holds. Effective parenting is the way to successful Parenting. The efficiency of parents in parenting depends upon the personal factors of parents, child characteristics, and environmental factors. When the resource demanded by the parents does not meet the need of the parents, stress arises. It’s typical parenting stress. Parenting stress occurs when a parent faces any outstrip of the three main components: insufficient parental functioning that affects the parent domain, characteristics of the child that affect the child domain, and social factors that affect the social domain (1). Compared to other stresses, parental stress is due to parenting practices (2).

When Parental stress is at a higher level, it affects the parent’s parental activities. It hinders them from using effective parenting strategies for child rearing and helping them develop adjustment practices (3). This high level of stress will result in less warmth between parents and children and lead to poor and unhealthy parenting (4). Four major variables that lead to stress are as follows, the reaction leads to stress, the determination of the mind whether it is an unpleasant event, strategies to minimize the unpleasant event and outer agent.

Attention deficit hyperactivity disorder is a prolonged disorder that affects children with inattention, impulsivity, and hyperactivity (5). Various research has been done on children with ADHD. Multiple studies explain the pain of rearing ADHD children. Podolski and Nigg (6) revealed that it is a tedious task to raise ADHD children, who display hyperactive behavior. Hyperactive children are mostly stubborn in nature, adamant about achieving their desire, and it is very difficult to satisfy them.

Mash and Johnston (7) state that the parents of children with ADHD (PCWADHD), experience high stress due to psychosocial reasons in their parenting role. The psychosocial reasons include home environment and their own in-built characteristics. Despite, there being enough evidence that PCWADHD undergoes depression and various other stresses, it is less fortunate that only a few studies have addressed these issues. The parents with ADHD children have severe stress compared to the parents with normal children. Befera and Barkley (8) explain that parental stress in the PCWADHD may be the outcome of marital dysfunction. Mash and Johnston (7) add to their view that parental stress of the PCWADHD may be due to their family size and the relationship between the children with ADHD and the other normal siblings which are often not positive. Their past negative life events may also increase their stress as suggested by Campbell et al. (9).

The parenting stress leads to the poor psychological well-being of the mother and affects their interaction with the child and the child’s development.

Various factors of parenting stress have been found in many studies. This case study provides an in-depth study considering all three major areas such as biological aspects, psychological aspects, and social aspects giving a holistic view of the reason behind the parenting stress and factors that can enhance psychological well-being.

## 2. Literature review

Abidin’s Parenting stress theory is a comprehensive framework that identifies various sources of stress in parenting, emphasizing the importance of the parent’s perception of stress. It highlights child characteristics, parent characteristics, and situational/contextual factors as key stressors, and it links parenting stress to the well-being of both parents and children (1).

Folkman and Lazarus (10) in their stress model emphasize that the reason for stress may be due to the communication that happens between the individuals or between family members and the environment. People use coping techniques to bring back their normal lives when they feel that they are caught in environmental stress. However environmental stress overshadows the individual’s coping techniques when the individual does not have ideas to counter the new demands or they have an incapable idea of overcoming the environmental stress. Studies by Carpenter and Steffen (11) also reveal that high stress may lead to depression, hormonal changes inside the body, tiredness, impatience, risk of heart disease, and high chances of ulcer. Stress is proportional to the behavior of each individual. Similarly, parental stress is proportional to the behavior of the family. The failure of the PCWADHD to find the coping techniques to handle their children with ADHD will lead to high parental stress. Parents’ stress and negative feelings are directly linked to parenting behavior, which will proportionately lead to the risk factor of a child’s mistreatment and neglecting (12). The parenting stress has a direct impact on parenting behavior, the quality of caregiving, and the emotional health of the child. The stress level exhibited by the parents may lead to a more confusing family environment, which may directly affect the behavior of the children with cognitive development (13). This parental stress can lead damage to the child’s emotional development, as children in houses where the parents show higher stress may experience low self-esteem and high nervousness (14).

However, very few studies have been done on how to minimize the stress level of these parents.

Stress levels are classified into different categories. Some stresses are external, and some are internal, some are acute whereas some are chronic. Many studies have concluded that a child who has ADHD is an external chronic stress factor, but it will affect the marital relationship of the parents and thus will be the primary reason for the internal chronic stress factor. Bodenmann and Cina (15) explained that this external chronic stress affects the communication between the parents which is considered as negative, and the adverse effect will be the breakup in their relationship. Byrne et al. (16) further detailed the various factors associated with ADHD children such as age, ADHD symptom level, and gender are the strongest factors that directly induce the internal chronic stress in the parents. Parental stress is high with the PCWADHD, and this stress level is higher when compared to the parents whose children are diagnosed with anxiety disorder. Abidin (2) explains in his parenting stress model, the reason why parents adhere to an inappropriate style of parenting. He explained that parenting stress will be determined by the level of mismatch between the needs of the PCWADHD and the resources around them.

Bandura (17) conveyed that the stress level in an individual is directly proportional to the individual’s capacity to control his/her emotions. The stress level will be high when the individual lacks control over his/her emotions. When children demonstrate stressful behavior, the negative emotions of the parents will shoot up and put them in a place where they will display inappropriate behavior or consider themselves incapable parents. Even when the stress of teachers was analyzed, it was found that teachers demonstrated higher stress levels when they were handling stressful students which resulted in poor management of the classroom. A recent study was done on PCWADHD children, and it was found that parents used irrational beliefs as coping techniques, they had guilty feelings and they exhibited low self-efficacy. Cappe et al. (18) conveyed that when parents see the handling of ADHD children as a challenge, they have satisfaction and feel they were in control of the situation. Bloomfield and Kendall (19) concluded that parenting stress depended on the ability of the parents to cope with the situation. When parents lack emotional control, their self-efficacy goes low. Pelletier and Brent (20) explained that parents should have the potential to give emotional and social support for their children to function effectively. O’Neil et al. (21) stated that parents with positive self-efficacy found appropriate ways to handle their children effectively. However, Primack et al. (22) stated that positive self-efficacy can be affected in the long run when handling ADHD children and reduce the confidence of the parents.

Belsky (23) in his theory proposed that for better and more efficient parenting practices, social support is one of the key factors. The quantity of social support actually received and perceived acts as a boosting system for successful parenting and when a mother lacks this kind of support that leads to inefficient parenting. The support system can be direct or indirect. Direct support is a means of rendering direct help and indirect helping can boost the mother’s parenting efficacy and esteem.

For affirmative mother–child connections, social support plays a vital role. When there is a lack of social support that leads to harsh behavior, emotional distance, rejecting and avoidance. Many studies reveal that good parenting and social support are interlinked to each other. Jennings et al. (24) identified that social support prevails in relationships where the mother and child have a positive interaction. When the children exhibit positive characterization, the parents receive high social support. Social support can minimize the stress incurred in the parents. McCurdy (25) stated that when the support system in the family decreases, stress is created which leads to aggressive parental behavior whereas when friends and partners show great support, the stress level decreases. A mother’s stress can be directly proportional to the level of support she gets from her surroundings, and that will determine the quality of interaction she will have with her infants. Crnic et al. (26) add that the relationship between the mother and the child also is one of the factors used to determine the social support of the mother. Having studied social support and its link with parenting behavior, it is also noted that social support does not reduce stress at all times. Ghate and Hazel (27) did an analysis with 1754 British parents to find the level of stress these parents showed when support shown to them in a formal and informal way. The parents revealed that formal support was less available when compared to the support received from informal sources and the parents declare that their stress level decrease with the formal support received. Byrne et al. (28) conducted a study with parents in regard to child maltreatment prevention programs. They studied the effects of support (both informal and formal) in relation to the timing (at the beginning or at the last stage of the program) in which the support was given to the parents. These studies showed a positive outcome when parents received informal support such as ideas about child development, some best practices for growing the child, the role of parents, etc., whereas, under formal support, the parents felt the positive impact. From these results, it is understood that when there is very minimal support, parents displayed greater stress whereas when parents were given support at each level, the stress level was minimal. Hence, it is known that when there is a lack of social support, parental stress is high. So, it is very important to investigate the relationship between social support and parenting stress to identify the steps that have a positive influence over good parenting practices. Being isolated from society and also lack of support from a partner, close friends or family are one of the major characteristics of vulnerable families. These studies found that families with a lack of social support trusted formal sources of support. The presence of experts in welfare organizations is proof of the trust in formal sources by the parents with less social support. Segal (29) explained that when children do not obey their parents, nor do they follow any rules and regulations, as their frustration threshold might be low. So parents of ADHD children should exhibit the highest quality of support between each other to rear their children. Parents who lack good communication between them are subject to higher conflicts which may eventually lead to dissatisfaction in the marriage life. Belsky and Kelly (30) and various other authors have documented that parents’ satisfaction level in their marital life is low when their child has ADHD. Similarly, we can find other studies that explain the stress and impacts of parents with ADHD children. Support from a partner is very much needed for a healthy well-being. The study done by WHO (31) and Kimuna et al. (32) on intimate partner violence (IPV) on women, suggest that 50 percent of South-east Asian women were facing such abuses. Indian women comprise of 16 percent of world’s women population. And 31 percent of Indian women face physical or sexual abuse by their intimate partner. However, National Family Health Survey states that there a drastic shift of intimate partner violence from 37 percent to 28.8 percent in the last 10 years (33).

The major challenge faced by Indian women is Domestic Violence (DV). This domestic violence in women has weakened their mental health as well as their physical health. a comparative study was conducted by taking reviews of studies based on domestic violence in Indian women in the past decade with the recent studies to understand the variation in the results. Hundred and thirty seven studies conducted in the past decade were considered for the study. The study gave the conclusion that there is minimal study of domestic violence on women over 50 years and there is no standard scale to study the domestic violence due to the diversified Indian culture. The study also informed that there is a gap in identifying the impact created on women’s physical health due to domestic violence (34). Indian epics have depicted women as strong and resilient forces. They show women as goddess (35). However, there are practical challenges both physically and mentally when it comes to the analysis of their daily lives.

As mentioned in the previous studies, there are studies that study the factors affecting a mother’s psychological well-being but a holistic study considering all the factors are missing. An in-depth case study is needed to identify the various factors affecting psychological well-being so that an intervention to address the issues can be developed and implemented. During the pregnancy, a mother will dream about their child. They dedicate their lives to bringing up their children. Their goals are not met when they find out their child has some disability or disorder. They face many challenges in caring for a child with special needs. A healthy life and a good mental state are needed for better psychological well-being. There are a lot of factors, external and internal, that affect the psychological well-being of the mother. There is a necessity to understand the factors that affect psychological well-being. As a result, this case study investigates the various factors that affect psychological well-being based on the Biopsychosocial model.

### 2.1. Biopsychosocial model

This model approaches health, based on three different factors such as physical health, psychological (that includes behavior, attitudes, coping skills, self-efficacy, self-esteem, and emotions), and social factors (socioeconomic status, relationships, peer relationships, and social support). According to this theory, all these factors play an essential role in health and disease. This model is proposed and used in health psychology, family therapy, clinical psychology, etc. George L. Engel (36, 37), a psychiatrist in 1977, developed this model and published it in 1980.

### 2.2. Objectives of the research

1. To better understand the factors affecting the psychological well-being of mothers of children with ADHD.
2. Using a Biopsychosocial approach, investigate the factors affecting the psychological well-being of the mother of a child with ADHD.

### 2.3. Research Question

1. What specific factors contribute to the impact on the psychological well-being of mothers raising children with ADHD?
2. How does the application of a Biopsychosocial approach aid in the examination of factors influencing the psychological well-being of mothers caring for children diagnosed with ADHD?

## 3. Methodology

This is a descriptive case study, and the purpose of this study is to understand the factors associated with the parenting stress and Psychological well-being of mothers of children with ADHD. The aim is to find the factors related to parenting stress and the mother’s psychological well-being based on three components such as biological, social, and physical, and to propose proper intervention.

### 3.1. Data collection procedure

The researcher approached a special school and requested permission to conduct a case study. A mother of a child diagnosed with ADHD was selected for the case study. A consent form was given to the mother for the willingness of the study. After obtaining the consent form, the mother was interviewed while the child was attending the therapy session. A rapport was created and questions were asked one by one. The mother was given enough time to think and answer the questions and the response was recorded and later verbatim was done. During the study, the mother was comfortable and opened up for all questions without hesitation. When the questions were related to her health and the child, she was a little reluctant and answered emotionally with so many pauses between the replies.

## 4. Parent history

### 4.1. Case study

The mother of the child was the firstborn in her family. She was a postgraduate and married her cousin when she was 23 years old (consanguineous marriage). They have been married for 14 years. After 6 years of married life, she delivered a baby boy. There was a complication during the child’s birth; the baby turned blue during the delivery and was under oxygen support for a few days. The milestones development of the child was normal. They lived in a joint family. When the child was 4 years old the mother noticed that the child was not listening to the instructions and was also very active, like not sitting in one place, climbing the windows, and jumping everywhere. The mother also found it challenging to handle the child in public places like malls, religious gatherings, family functions, etc. Also, the child found it difficult to concentrate in class and pay attention to the instructions in school. The special educator in the school suggested the parents have a check with a clinical psychologist. After examining the child, the clinical psychologist diagnosed the child with Attention Deficit Hyperactivity Disorder, which has impulsivity, hyperactivity, and attention problem characteristics. The child was asked to undergo therapies and attend a special school for therapy programs. There was a history of ADHD in the family. One of the paternal uncles of the child has also been diagnosed with ADHD and was under treatment. Parents and teachers found the reason behind the child’s poor academic skills, behavioral skills as well and social skills. The mother faced discrimination, social stigma, and isolation from family, friends, and society. The mother of the child felt less competent and stressed in handling a child with ADHD. The mother lacked knowledge about the disorder and had not gone for any intervention program to strengthen her capacity to handle the hyperactive child.

## 5. Presenting problem

The child was diagnosed with attention deficit hyperactive disorder. ADHD is a neurodevelopmental disorder that can affect a person’s ability to focus, control their impulses, and manage their behavior. It can indeed lead to challenges in academic, behavioral, and social aspects of life. Due to the disorder, the child lacked academic, behavioral, and social skills. Parents faced discrimination, social stigma, isolation, and lack of support from the family and society. The child’s mother felt less competent in handling the child and was stressed. The mother often felt worn out and was not equipped to take care of her child and felt frustrated. Parents should seek support and guidance from professionals who specialize in ADHD. Parent training programs can teach effective strategies for managing a child with ADHD and help parents feel more confident in their parenting abilities. So the factors associated with these problems should be identified in order to develop an intervention module addressing all these factors to enhance the psychological well-being of mothers.

## 6. Results

Table 1 shows the factors identified through the case study by the biopsychosocial model. In the predisposition factor, the biological factors are poor health and genetic factors, the psychological factors are low self-efficacy and low self-esteem, and the social factors are discrimination and social stigmatization. In the Precipitating factors, the biological factors are Lack of sleep, the psychological factors are Loss of a loved one, and the social factors are Economic status. In the Perpetuating factors, the biological factors are Lack of knowledge, the psychological factors are Guilt and instability of mind, and the social factors are Lack of independence and lack of empathy from others. In the Protecting Factor, the biological factors are Good health, proper sleep, and capacity building, the psychological factors are Self-esteem, and parenting self-efficacy, and the social factors are Social support from family and friends.

**Table 1:** Bio psycho socio factors.

	Biological factor	Psychological factor	Social factor
Predisposition factors	Poor health, genetic factors, parenting stress	Low self-efficacy and low self-esteem, emotional regulation	Discrimination and social stigmatization, Marital and Family Stress
Precipitating factors	Lack of sleep	Loss of a loved one, Lack of Personal Time	Financial Strain
Perpetuating factors	Lack of knowledge	Guilt, instability of mind, self blame	Lack of independence, lack of empathy from others, Access to Resources, domestic violence
Protecting factor	Good health, proper sleep, capacity building	Self-esteem, parenting self-efficacy	Social support

Raising a child with ADHD can be challenging and can have a significant impact on the psychological well-being of mothers. The major factors identified are as follows.

1. Parenting Stress: The daily challenges of managing a child with ADHD, such as impulsivity, hyperactivity, and inattention, can lead to increased parenting stress. The Mother constantly worries about her child’s well-being, education, and future, which can take a toll on their mental health.
2. Stigma and Social Isolation: The mother experiences stigma and judgment from others who do not understand ADHD. This can lead to feelings of social isolation, as she withdraws herself from social activities or relationships to avoid criticism or discomfort.
3. Financial Strain: The costs associated with ADHD, including therapy, medication, and educational support, can be significant. Managing these expenses can add financial stress that affects the mother’s mental well-being.
4. Disrupted Daily Routine: Children with ADHD often have erratic sleep patterns and difficulties with routines. This disrupts the mother’s daily life, making it harder to manage her own self-care and maintain a healthy work-life balance.
5. Emotional Regulation: Children with ADHD may have difficulty regulating their emotions, leading to frequent outbursts or meltdowns. Constantly dealing with these emotional challenges also be emotionally draining for mothers.
6. Marital and Family Stress: Raising a child with ADHD can strain marital and family relationships. Conflicts may arise over parenting strategies or the division of responsibilities, adding to a mother’s stress.
7. Guilt and Self-Blame: Mothers may blame themselves for their child’s ADHD or feel guilty about their parenting abilities. These negative emotions can contribute to depression and anxiety.
8. Lack of Personal Time: Constant caregiving responsibilities may leave mothers with little time for themselves, leading to burnout and a decline in mental well-being.
9. Health Issues: The chronic stress associated with raising a child with ADHD can lead to physical health problems, further impacting psychological well-being.
10. Access to Resources: The availability and accessibility of resources, such as support groups, therapy, and educational services, can vary widely depending on where a mother lives. Limited access to these resources can exacerbate the challenges of raising a child with ADHD.

## 7. Discussion

### 7.1. Predisposing factors

The factors that are very vulnerable that increase the risk of the problem are said to be predisposition factors.

#### 7.1.1. Biological factors

After the child’s birth, the mother underwent another surgery that led to her poor health. Mother felt weak and tired most of the time as she ran behind her child. The mother was not a very healthy person, and also she was in her pre-diabetic stage. The mother was anemic and taking medication for that. There is a chance of the hyperactive problem running in the family. The paternal uncle also had the same issue and the genetic factors may also been the cause of the disorder.

*“I have no strength to care for my child; whenever a motor drives him, I feel exhausted. Sometimes I feel that because of my inability, everyone blames him and tells me he is not listening to others.”*

Health is essential for healthy psychological well-being, mental health, and happiness. Health is a primary source of mental well-being [Jinmyoung (38)].

#### 7.1.2. Psychological factors

The mother lacked confidence in handling her hyperactive and in-attentive child. She felt she was not a good mother and incapable of being a mother. The mother of the child felt her mother was a better mother and had more knowledge of handling a child. When asked whether she could guide another mother regarding parenting skills, she said she needed to be more confident to guide another mother as she lacked self-confidence. Her self-efficacy and self-esteem were very low.

*“I do not know how other mothers effectively handle their children. I should learn strategies from others. I may not be able to guide new mothers as I lack the knowledge of effectively handling children, but maybe my mother can guide me; she is the best mom when I think about her parenting skills; I feel I am nothing and have to learn a lot.”*

Parenting a child with hyperactivity and inattention is a challenging process. Parents should capacity themselves to handle such a child. Parents may face low self-confidence and self-esteem when handling a child with a developmental disorder.

#### 7.1.3. Social factors

The mother was a homemaker and needed to take care of the child’s needs. She quit the job because she could not manage her work, taking care of the child, and house chores. The family income was insufficient to care for the child and to take him to different intervention programs. Odam et al. (39), describe the importance of early intervention for the children with special need and toxic effect of stress. Also, they live in a joint family where the elders in the house had different rules and it was difficult for the child to follow the rules. The mother was blamed every time the child disobeyed the rules. This situation created tension in the family, leading to stress for the mother.

*“I wanted to work and be independent. Nevertheless, I quit my job to take care of my child. For every expense, I need to ask my in-laws or my husband for money. I wanted to put my son in a school that had a special educator and shadow teacher, but as it demanded more fees, my family members rejected it, and I could not afford it by myself, so I did not admit my child to the school even though I wanted to put my son there.”*

The mother faced discrimination and stigmatization by the people around her. The mother felt that society believed that due to her mistakes and the sin she had committed, she had an ADHD child. Also, she thought that the child’s disorder was due to her carelessness and poor eating habits. Stigmatization and discrimination can lead to poor psychological well-being (40).

### 7.2. Precipitating factors

Precipitating factors are the factors that are the reason behind health issues or illness. The factors act either positively or negatively toward the disease.

#### 7.2.1. Biological factors

The predisposing factor of the mother alone does not contribute to parenting stress. There are certain factors that contribute as a stressor and lead to parenting stress. Sleep deprivation and lack of sleep are the main factors leading to stress. Also, the child needs more attention and care from the mother to practice his daily routine and school work. So, the mother has to give as much care as she gave when the child was an infant. The child is hyperactive, so the mother has less time to rest during the daytime. Practicing the child for his daily activities is time-consuming, and the mother faces energy loss, leading to tiredness.

*“I wanted to sleep during afternoon hours as I get up very early in the morning, but I will not sleep as I need to be with my son. If I sleep, he will drag all things from the kitchen, and I need to work harder to place them back.”*

These factors are not one-time events and appear multiple times. This affects the psychological well-being of the parents. As health is affected, it also affects the mental state directly. These factors act as a threshold (41).

#### 7.2.2. Psychological factor

The mother of the child lost her father a year back. Her father supported her financially. Also, she was forced to change the house due to financial issues. The mother had a neighbor with whom she shared all her distress and grief. Because she could not share her worries with anyone near her new place. After coming to a new place, the mother felt her stress level became high. As support from her parents stopped, the mother felt helpless. Also, her spouse is very hard with her sometimes as she could not finish her house chores on time.

*“I felt lost when I lost my father. He was supporting me financially. Now I depend on my husband and in-laws for every single expense. I was hesitant to ask, so I stopped having wished for myself. Also, I had a perfect friend who hears all my worries without judging me.”*

Loss of loved ones, especially the person who supports, believes, and helps are lost, then the loss is incredible. It will take time to come out of the trauma. This will affect Psychological well-being. The death of a family member who was the source of support and was unexpected leads to a high burden and stressful experiences. That trauma experience can lead to poor mental well-being (42). Misunderstanding in families, the conflict between father and mother that even leads to harsh behavior with physical assaults also results from parenting stress (3).

#### 7.2.3. Social factor

The income of the family fell in lower socioeconomic status. They found it challenging to afford a particular school as the therapy for the child costs high. The mother was devastated when the child was refused admission to a non-special school as the child was very hyper, and the school did not have a special educator to care for the child properly. The parents were forced to put their child in a school with a special educator. However, the fees were not affordable for them. Moreover, the mother had to take care of the child, and she could not go for a job; this also frustrated the mother.

*“I wish all schools should have a shadow teacher or special educator to take care of the children with special needs. Also, the government can open special schools that will be more convenient and affordable for parents like us. I was broken when my son’s admission was rejected because of his disorder.”*

Special therapies are higher-end and may not be affordable to the parents. Also, only some schools have special educators to accompany the child and give special training. Parents feel discouraged when they cannot afford to meet their child’s needs. It is essential to put into practice the knowledge gained in parenting (43). This helps transform a child’s cognitive behavior into social–emotional behavior. Studies reveal that a mother with absolute knowledge about child rearing engages their children with books and study materials that enhance their children’s reading, talking, and narration abilities. Mothers who lack knowledge in child-rearing may not focus on enhancing their child’s abilities. Engaging the children with activities will enhance the child’s abilities and satisfaction for the mother [(44), p. 222].

### 7.3. Perpetuating factors

The factors that exacerbate the problem in a worsening way are the perpetuating factors.

#### 7.3.1. Biological factors

Parenting was a challenging as well as demanding process. There is a lot to learn for better parenting. Parenting a child with special needs is very challenging. The mother may not know what is happening with the child and how to handle it. First, accepting that the child has some problem is the most challenging thing for the mother. Every mother has the most significant dream about her child. The mother should gain knowledge, strategies, and techniques and build her capacity to handle the child when it is not attentive and when the child is very hyper. This mother did not attend any training program or know the reason behind the child’s problem. She was unaware of what was happening to the child. Due to her incapability of handling a child with hyperactivity and attention problems, she felt frustrated, incapable, and a failed mother. The mother did not use coping mechanisms like meditation or entertainment to calm herself down from stress.

*“This is my first experience. I never heard about ADHD before. I do not know what to do and how to do it. That’s why I put him in a special school. He is in school for 8 h and drives me mad at home. I feel guilty as I could not care for my child well. I feel I am a failed mother. Instead of supporting me, my family blames me for everything that goes wrong.”*

A child needs to be cared for in a way that the child grows up with ability, which promotes mental health and emotional well-being. A child should be competent enough to socialize in society (45). A child relies on the mother very much for the development. The mother must equip herself to train herself and also the child. Lack of knowledge leads to a thought of failure (46).

#### 7.3.2. Psychological factor

The guilt of not taking care of the child leads to stress. The mother had no control over her emotions and felt worn out. She was exhausted most of the time. Sometimes she thought she would die soon because of uncontrollable feelings and emotions. She forgot things often and fell sick. She crossed the stage where she could not do any work and depended on others for everything. She sometimes had to fight her emotions so that she could be expected. Doing daily chores had been a burden for her.

*“There was one time when I felt my life was going away. Like every mother has a dream about their child, I also had a dream. But I was devastated when I felt my child was not like another child. My whole world turned upside down. My dreams shattered. It took many days for me to accept the fact, and during those days, I was like an unhealthy person. I seek help from everyone. I thought ill become weak, and I could not become normal like before.”*

Mothers end up with emotional tiredness, physical weakness, and mental breakdown when they are stressed by taking sole care of the child. That is caused due to excessive demand and restriction that leads to an impact on their psychological well-being (47).

#### 7.3.3. Social factor

A supporting system is very much needed for a person’s well-being. It not only gives comfort physically, but it also gives comfort mentally. The mother was blamed for all the discomfort the child created at home and during the family meets. She does not receive support from her husband, especially for the child. The mother lacked empathy from her family, friends, and neighbors. The mother had to stay home with her child while others went on vacation, temple, or for family gatherings. The mother felt left out and isolated even though many people surrounded her. Everyone in the family thought caring for the child was only her responsibility. The mother had to sacrifice her job to look after the child. In that way, she lost her independence. The mother stopped going out to family gatherings, wherein more people gathered. The mother also stopped all the entertainment like going to a movie, shopping in malls; friends meet so on because she cannot leave the child and there is a lack of support to babysit the child. The daily routine of women was to finish house chores, take the child to school, pick up her child, and so on. It was more like monotonous work. The mother felt that no exciting thing was happening. When she tried to share her worries with her friends, they also showed no interest. The mother felt that she was losing the support system one by one. She was affected more when her partner was violent towards her.

*“I worry for myself; no one can care for my child if I am not there. That is my biggest fear of my life. I see that no one is interested in caring for my child. Even my family members are not willing to spend time with him. Instead, they blame me if anything goes wrong. Only my mother sometimes helps me. As she is old, I cannot depend on her more. I cannot make decisions for my child as I need to depend on others financially. I used to think I would put him in many intervention classes if I went for a job. I sacrifice my entertainment, outings, and even some rituals to care for my child. To me, he is everything.”*

The mindset of present-day parents in raising their children differs between men and women. The research conducted on the presumption of the women in the involvement of men in child-rearing showed that women felt superior in their involvement in rearing their child compared to the role of the child’s father (48). Women are affected more psychologically due to the violent activity of their partners (49). Bodenmann (50) stated that married couples undergo external stress for reasons such as financial conditions or reasons that are outside their control. Bodenmann and Cina (15) stated that internal stress between the married couple may be due to poor communication and the level of cooperation between them. Johnson et al. (51) explained that stress in parents created by children is external, as the stress is not caused because of the conflicts between the married couple but it is due to the children. But this external stress will ultimately lead to conflict between the couple and thus will result in internal stress. Erel and Burman (52) termed this event as “Spillover effect” which means the emotion of one person will influence the emotions of the people around them. Here, the child’s stress influences the parents. Acute stress is a cause of a single event and hence concludes it as temporary. Johnson et al. (51) state that chronic stress is a result of stable events and conclude that this stress will be perpetual.

### 7.4. Protecting factor

The factors that prevent the problem or slow down the severity of the problem and strengthen the person are protecting factors.

#### 7.4.1. Biological factor

While looking upon all the factors that lead to the poor psychological well-being of the mother, it is seen that the mother lacked the knowledge of how to handle her child with attention problems and hyperactivity. This lack of knowledge leads to a poor understanding of the child’s behavior and parenting stress. Also, the mother did not get proper rest and sleep; her improper lifestyle made her feel weak and worn out. Poor health maintenance leads to stress and weakness. The mother could not accept that her child was having a problem. She was feeling down and worried. She did not take any measures to bounce back and be normal. The mother might be low in resilience. It can be seen that the characteristics of the child and mother result in parenting stress and low parenting self-efficacy.

#### 7.4.2. Psychological factor

From the case study, it can be understood that the mother did not adopt any coping mechanism to handle her stress and depression. Also, she wasn’t trained to improve her self-esteem and self-efficacy. Improving their self-efficacy will boost their self-confidence and increase their well-being.

The mother also lacked a positive insight into herself. She believed that the suffering was due to some curse. It is a must to increase her love for herself so she can feel positive and light, boosting her self-care. The studies of (53) reveal that children with mental growth deficiency exhibit behavior problems to a more significant extent and hence develops mental disorder. Also, studies have shown that children with mental disorders have exhibited both internal and external behavior problems. A longitudinal sample study was conducted by Baker et al. (54) on children with developmental delays, and they found that 26.1% of the children showed clinical levels of behavior problems and 8.3% of children exhibited typical development. Herring et al. (55) states that children with developmental delays are an important factor in determining the relationship between the child and the parents. This can lead to parenting stress.

#### 7.4.3. Social factor

From the answers shared by the mother, it was self-evident that the mother lacks social support. The mother lacks support from her family, relatives also from friends and faces social stigma when she goes out to family functions and to society. The child was denied admission to schools without a special educator.

When the mother lacks support from others, she feels it’s a burden to carry all the responsibility by herself. That leads to frustration and low psychological well-being. Also, as the child has been rejected and not accepted by his peers and other family members, it leads to poor well-being of the child, seeing the mental suffering of the child, the other to suffer mental agony (56). The mother lacks income and that restricts her from making decisions and spending money. This is a sign of poor interpersonal relationships. If the mother gets support financially, emotionally, and physically, she might experience good perceived social support, and that might increase her well-being (57). Also, the mother might feel comfortable and courageous if the mother has a good interpersonal relationship. Mothers and fathers who are a risk in psychosocial are the people who have problems in their surroundings and affect the development of their children (28). All the above factors lead to stress in the parents, especially mothers and this affects parental functioning directly or indirectly (58). Positive psycho-social impact helps to improve the strategy-making ability for effective inclusion (59).

The biopsychosocial approach is a comprehensive framework used in healthcare and psychology to examine and understand the factors that influence an individual’s well-being. When applied to the examination of factors influencing the psychological well-being of mothers caring for children diagnosed with ADHD, this approach considers a wide range of biological, psychological, and social factors that interact with and impact the mother’s mental health. By considering all the factors together, the biopsychosocial approach provides a more holistic understanding of the mother’s experience. It recognizes that addressing psychological well-being involves addressing multiple facets of a person’s life.

## 8. Conclusion

A mother plays a crucial role in a child’s development. A healthy childhood builds confidence and courage in the child. According to the culture in India and especially in Tamil Nadu mother is very much responsible for child care. Society expects the mother to be responsible for child-rearing, and society blames the mother if anything goes wrong (60).

The child’s father concentrates on his job and takes less responsibility in child care. Based on the case study, it is identified that the mother is the only person who cares for the child who has ADHD. She sacrifices her career, enjoyment, and lifestyle to care for her child. She faces social stigma, negligence, avoidance, and discrimination. She lacks support from her family and friends. The mother did not equip herself to gain knowledge about her child’s disorder. She lacks the ability to handle the child, especially when the child is hyperactive and not attentive. She feels low in spirit, and her confidence level is very low, especially when handling the child. Her resilience power is also deficient. All the above factors lead to parenting stress and poor psychological well-being. The mother can attend a training program that enhances self-efficacy and reduces parenting stress. The mother can do positive parent training, mindfulness training, cognitive behavior therapy, parent-mediated social communication therapy, and meditation techniques to enhance her psychological well-being. Based on the study an intervention module can be developed with sessions like psychoeducation, tips to handle a child with attention problems and hyperactivity, self-perception, how to resolve relationship conflicts, meditation techniques, etc.

### 8.1. Limitations of the study

This case study is based on an in-depth study of one mother of a child with ADHD. So the factors found were based on one mother’s experience and perception. Factors influencing mothers of children with ADHD can be influenced by various circumstances, including the child’s age, presence of comorbidities, and individual family dynamics. It’s essential to consider a broader range of perspectives to gain a comprehensive understanding of the challenges and factors that influence the well-being of the mother.

### 8.2. For further research

The factors affecting the psychological well-being of mothers were identified in the study. An intervention module can be developed to address these factors to check whether psychological well-being is increased after the intervention program.

## Data availability statement

The raw data supporting the conclusions of this article will be made available by the authors, without undue reservation.

## Ethics statement

Ethical approval was not required for the studies involving humans because it was conducted on adult person. The studies were conducted in accordance with the local legislation and institutional requirements. The participants provided their written informed consent to participate in this study. Written informed consent was obtained from the individual(s) for the publication of any potentially identifiable images or data included in this article.

## Author contributions

JA: Conceptualization, Writing – original draft. MR: Writing – review and editing.
